# Multi-View Feature Enhancement Based on Self-Attention Mechanism Graph Convolutional Network for Autism Spectrum Disorder Diagnosis

**DOI:** 10.3389/fnhum.2022.918969

**Published:** 2022-07-15

**Authors:** Feng Zhao, Na Li, Hongxin Pan, Xiaobo Chen, Yuan Li, Haicheng Zhang, Ning Mao, Dapeng Cheng

**Affiliations:** ^1^School of Computer Science and Technology, Shandong Technology and Business University, Yantai, China; ^2^School of Management Science and Engineering, Shandong Technology and Business University, Yantai, China; ^3^Department of Radiology, Yantai Yuhuangding Hospital, Yantai, China

**Keywords:** resting-state functional magnetic resonance imaging (rs-fMRI), graph convolutional network (GCN), pooling operation, feature enhancement, autism spectrum disorder (ASD)

## Abstract

Functional connectivity (FC) network based on resting-state functional magnetic resonance imaging (rs-fMRI) has become an important tool to explore and understand the brain, which can provide objective basis for the diagnosis of neurodegenerative diseases, such as autism spectrum disorder (ASD). However, most functional connectivity (FC) networks only consider the unilateral features of nodes or edges, and the interaction between them is ignored. In fact, their integration can provide more comprehensive and crucial information in the diagnosis. To address this issue, a new multi-view brain network feature enhancement method based on self-attention mechanism graph convolutional network (SA-GCN) is proposed in this article, which can enhance node features through the connection relationship among different nodes, and then extract deep-seated and more discriminative features. Specifically, we first plug the pooling operation of self-attention mechanism into graph convolutional network (GCN), which can consider the node features and topology of graph network at the same time and then capture more discriminative features. In addition, the sample size is augmented by a “sliding window” strategy, which is beneficial to avoid overfitting and enhance the generalization ability. Furthermore, to fully explore the complex connection relationship among brain regions, we constructed the low-order functional graph network (Lo-FGN) and the high-order functional graph network (Ho-FGN) and enhance the features of the two functional graph networks (FGNs) based on SA-GCN. The experimental results on benchmark datasets show that: (1) SA-GCN can play a role in feature enhancement and can effectively extract more discriminative features, and (2) the integration of Lo-FGN and Ho-FGN can achieve the best ASD classification accuracy (79.9%), which reveals the information complementarity between them.

## Introduction

Brain disease is regarded as a public health challenge with an alarming proportion (Yao et al., 2021). Among them, autism spectrum disorder (ASD) is a complex genetic heterogeneous neurological disease with high incidence rate, usually coexisting with other diseases (Lord et al., 2020; Hiremath et al., 2021). According to the latest report of the Centers for Disease Control and Prevention, there is one autistic in every 44 American children (Maenner et al., 2021). So far, there is no effective method to completely cure autism, and the rehabilitation treatment of autism is a lifelong training, which causes heavy economic burden to the families and society (Eslami et al., 2019; Zhao et al., 2020). Thus, early diagnosis and intervention of autism is of great clinical and social value (Zhao et al., 2018; Wang et al., 2019; Hiremath et al., 2021).

Resting-state functional magnetic resonance imaging (rs-fMRI) based on blood oxygen level dependent (BOLD) signal imaging is an important tool to explore brain mechanism and pathology (Chen et al., 2016; Gan et al., 2021). Rs-fMRI can realize non-invasive study of brain function high spatial resolution, which cannot only reflect the local spatial function information of the brain, but also maintain detailed functional connectivity maps of the brain (Zhi et al., 2018). Rs-fMRI has been widely used to detect and characterize functional interconnection among different region of interests (ROIs), revealing potential patterns to distinguish between patients and healthy controls (Yao et al., 2021).

Currently, many extracting feature methods based on rs-fMRI are presented from different angles for disease diagnosis. Generally, they can be divided into two categories.

The first category focuses on extracting the features from each brain region without considering their connection relationship to each other; that is, the time-domain and frequency-domain features of each brain region of interest (ROI) are directly extracted based on the original BOLD. For example, Sartipi et al. (2018) proposed that based on generalized autoregressive conditional heteroscedasticity, the time-frequency sub-bands obtained by decomposing the brain ROI of subjects were extracted to diagnose ASD; Sidhu (2019) proposed the local linear embedding method, and the information measure of potential neuronal activity was extracted from BOLD time series for disease classification; Easson and McIntosh (2019) measured the variability of resting BOLD based on mean square continuous difference of time series and evaluated its complexity based on sample entropy to find predictors of ASD diagnosis. The above methods rely on brain ROIs, and the pathogenesis of brain diseases is explored by measuring the activities of various brain regions to assist the diagnosis of ASD. However, such methods ignore the connections among brain ROIs. Since the brain is a complex biological information system, each brain area is not isolated, but is interconnected on multiple spatial and temporal scales, working in coordination, the relationship among brain areas contains rich useful information for disease diagnosis.

The second category is committed to explore the functional connectivity among ROIs, through constructing functional connectivity (FC) network and conduct classification according to the differences in FC patterns among brain ROIs. For example, Zhang et al. (2020) learned multi-view features with multiatlas-based FC network to improve MCI diagnosis; Zhou et al. (2018) enhanced the high-order FC network based on regularization learning framework to identify the patients with MCI and ASD; Zhao et al. (2021) extracted the temporal-invariant properties contained in low-order and high-order dynamic FC networks based on the central moment method, revealing that different networks can identify the fingerprint of the autistic brain at different connection levels; Wang N. et al. (2022) identified ASD using multi-point clustering and nested feature extraction of rs-fMRI. Despite the effectiveness of the above methods captures features, they ignore the features of each brain ROIs and do not organically integrate the features of nodes (each brain region) and edges (the connection relationship among brain regions), and thus, they cannot extract relatively comprehensive and powerful discriminative features. Therefore, how to enhance the node features through the connection relationship between nodes and realize the organic combination of nodes and edges is an important research topic for ASD diagnosis.

In recent years, graph convolutional network (GCN) has achieved great success in dealing with non-Euclidean spatial data in the form of graph data (Xu et al., 2020, 2022; Li L. et al., 2021; Song et al., 2021; Ghorbani et al., 2022). GCN is able to automatically extract feature of brain network through an end-to-end manner, which is used for the recognition and classification of brain disease (Wang et al., 2021; Zhu et al., 2022). Specifically, GCN has the capability of transmitting, aggregating, and updating the node information in the graph, which can use the connection relationship of the nodes in the graph to enhance the node features, explicitly capture the node information and topology of the graph network, and mine useful brain connection network patterns for disease classification (Ktena et al., 2018). For example, Cao et al. (2021) used DeepGCN to identify ASD from multi-site resting-state data; Wang Y. et al. (2022) conducted diagnosis of ASD based on multi-spectral convolution network and ensemble learning. However, the existing GCNs still have some drawbacks listed as following when applied to brain FC networks.

(1) For high-dimensional small sample, GCN may not work well. A large number of training samples are often required for GCN training to avoid overfitting, which is hard to be satisfied in the single site of medical imaging. For example, the Autism Brain Imaging Data Exchange (ABIDE) database consists of 17 international imaging sites, of which New York University site has the most rs-fMRI data, including only 92 subjects (Di Martino et al., 2014). To solve this problem, previous studies usually collect data from multiple sites and put multiple data sources together (Cao et al., 2021). However, the problem of inconsistent parameters of multiple data sources may affect the learning performance of GCN.

(2) GCNs generally focus on the node information in the brain function connectivity network, but ignore the network topology and lack efficient graph pooling operation. GCN for graph classification mainly predicts the class labels of the whole graph by combining the learning methods of graph convolution layer, graph pooling layer, and readout layer (Pan et al., 2015, 2017; Ying et al., 2018; Zhang et al., 2018). Among them, the graph volume layer is responsible for accurate high-level node representation, whereas the graph pool layer learns the hierarchical representation of the network and reduces the parameters (Lee et al., 2019).

(3) In terms of graph network construction, previous studies usually start from a single level and then to extract features. They ignored two facts in the setting of node feature matrix and adjacency matrix of initial graph network. First, in the selection of node features, the FC network reflecting the connection relationship between nodes is considered, while ignoring the original blood oxygen signals in each brain region (Song et al., 2021); second, in the topological structure of the graph network, the connection between the two brain regions is considered, whereas the deep connection among nodes is ignored. For the ease of understanding, we use social networks as an analogy. Each brain region is regarded as an individual. In addition to its own unique features, each individual also has his/her own friends. Previous studies have focused on the interaction between individuals and their friends, but ignored individual unique features and the interaction between the circle of friends.

To handle the above issues, we propose a novel multi-view brain network feature enhancement method based on self-attention mechanism graph convolutional network (SA-GCN). Specifically, we first adopt the “sliding window” strategy to expand the sample size, i.e., the whole rs-fMRI time series is divided into multiple overlapping sub-segments by “sliding window” methods, and each sub-segment constructs a graph network, so that more samples are generated from one rs-fMRI time series for improving the overfitting problem caused by small samples and solved the problem of inconsistent parameters of multiple data sources in previous studies, making the experimental performance more stable; Then, we facilitate the graph pooling operation *via* self-attention mechanism in GCN, which considers both node features and network topology, and can filter useless informatics, leave more advanced, deeper and more discriminative node features; Furthermore, two different levels of FGN, i.e., Lo-FGN and Ho-FGN, are constructed from fMRI data to comprehensively capture the information contained in the brain network. The Lo-FGN reflects the changes of original BOLD in each brain region in terms of node features, and the connection strength between two brain regions in terms of network structure. The Ho-FGN reflects the interaction among brain regions in terms of node features and the deeper connection among multiple brain regions in terms of network structure. Finally, the multi-level features extracted based on SA-GCN are fused to realize the information complementarity between features, which is helpful to identify brain diseases, such as autism.

The rest of this article is organized as follows. In the Introduction section, we introduce related works of GCN in graph-level processing tasks. In the Proposed Methods section, our approach is described in detail, including data augmentation, self-attention pooling operations, and network construction. In the Experiments part, we present the experimental results, discuss different feature evaluation methods, and compare our strategy with other state-of-the-arts. Finally, conclusions are given.

## Introduction of Graph Convolutional Network

At present, GCN is one of the favorites in graph data learning tasks, which has wide applicability and is suitable for nodes and graphs with any topological structure (Rubinov and Sporns, 2010; Zhou et al., 2020; Li X. et al., 2021). Here, we focus on GCN for graph level tasks. GCN is essentially Laplacian smoothing on the network, which takes the weighted sum of neighbors and self-expressions of each node as the feature (Parisot et al., 2018; Shao et al., 2021).

**FIGURE 1:**
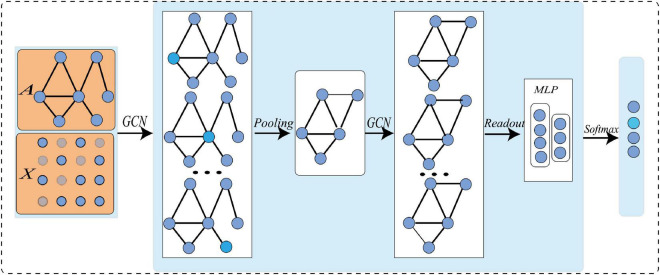
GCN with pooling layer and readout layer for graph level tasks.

The typical architecture of graph-level task GCN is shown in Figure 1. Firstly, the node feature matrix and adjacency matrix of the initial graph network are input into GCN; Then, the graph convolution operation is conducted at each layer to characterize the local structure of the node, and extract high-level node representation (Gu et al., 2021); After that, the graph pooling operation is facilitated to learn the hierarchical representation of the network (Henaff et al., 2015); Finally, with certain loss functions, gradient back propagation is used to train the network. All convolution layers share the same adjacency matrix. To increase non-linearity, the *ReLU* activation function is added after each layer. The iterative update operation can be expressed as:


(1) $$X^{\left(l+1\right)}=R\,e\,L\,u\,\left(p\,o\,o\,l\,i\,n\,g\,\left({\hat{D}}^{-1/2}\,\hat{A}\,{\hat{D}}^{-1/2}\,X^{\left(l\right)}\,W^{\left(l\right)}\right)\right)$$

where *A* ∈ *R*^*n*×*n*^ is an adjacency matrix, which defines the connection between nodes, and in an undirected graph *A*_i,j_ = *A*_j,i_. *I*_n_ϵ*R*^*n*×*n*^is an identity matrix, and$\hat{A}=A+I_{\text{n}}$. ***D*** is a diagonal matrix, *D*_i,j_ represents the degree of the *i*−*th*node and${\hat{D}}_{\text{ii}}=\sum_{\text{j}}{\hat{A}}_{i\,j}$. ***W*** is the trainable weight, *X*^(*l*)^is the *l-th* node feature matrix, where *X*^(0)^is the original node feature matrix. The complete GCN can be obtained after L iterations of training ^(^Parisot et al., 2018; Gu et al., 2021).

Although GCN can do feature extraction and enhancement by considering both nodes and edges in the graph network, it cannot be directly applied to our task. Specifically, there are two limitations: (1) The performance of GCN heavily depends on training samples, and our sample size is small. To solve this problem, we must expand the sample size; (2) Previous graph pooling methods either only consider the topology of graphs, or have high spatial complexity (Defferrard et al., 2016; Rhee et al., 2017; Cangea et al., 2018; Ying et al., 2018; Zhang et al., 2018). To reduce the learning parameters and computational complexity, it is necessary to improve the graph pooling operation. To tackle these two problems, we give the corresponding solutions in the proposed methods.

## Proposed Methods

**FIGURE 2:**
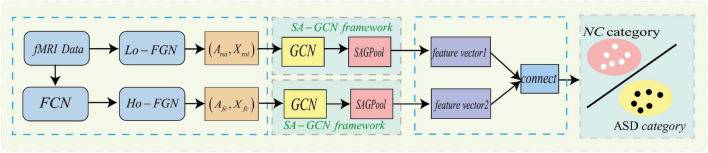
Overall frame diagram, where *FCN*: functional connectivity networks;*Lo*−*FGN*: Low-order functional graph network; *Ho-FGN*: *High*−*orderfunctionalgraphnetwork*; *A*_*roi*_ represents the adjacency matrix of Lo-FGN; *X*_*roi*_ represents the node feature matrix of Lo-FGN, others are the same as above.

To make GCN adapts to our task and data, we propose a novel multi-view brain network feature enhancement method based on GCN with self-attention mechanism (SA-GCN). The overall framework of our model is illustrated in Figure 2. To be specific, we first use the “sliding window” strategy to enlarge the sample size, and the low-order functional graph network (Lo-FGN) and high-order functional graph network (Ho-FGN) are constructed; Then, the pooling operation of self-attention mechanism is added to the GCN architecture to extract more discriminative features; Finally, the Lo-FGN and Ho-FGN are integrated based on SA-GCN to capture more comprehensive and discriminative features. Figure 2 illustrates the overall framework of our model.

### Data Augmentation

**FIGURE 3:**
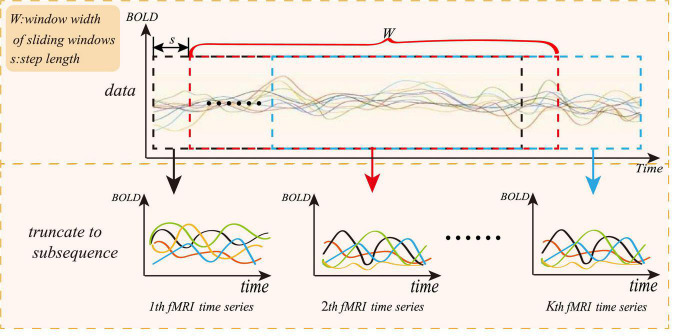
Sliding window method diagram.

To solve the small sample size of rs-fMRI data, we adopt a “sliding window” method for data augmentation, as shown in Figure 3, where the abscissa represents the acquisition time of the fMRI time series, and the ordinate represents the blood oxygen signal in the brain region. For each subject, the average rs-fMRI time series of all voxels in the *i-th* brain ROI is defined as follows:


(2) $$x_{\text{i}}=\left(x_{\mathrm{i1}},x_{\mathrm{i2}},\ldots ,x_{\text{iN}}\right)\left(i=1,2,\ldots ,R\right)$$

where *R* is the total number of regions of interest and *N* represents the total number of image volumes during rs-fMRI scanning. The whole rs-fMRI time series is divided into *K* overlapping sub-segments. Each sub-rs-fMRI time series can build a graph network. The value of *K* is calculated according to the following:


(3) K=((M-W)/s)+1

where *M* is the length of the entire rs-fMRI time series, and *W* is the length of the sliding window. To ensure that each sub-window owns relatively more rs-fMRI time information, *W* can be set to a relatively large value, and *s* is the step length of each slide of the sliding window. Therefore, the augmentation of the experimental data can be achieved through the “sliding window” method.

### Pooling Operation for Graph Classification

**FIGURE 4:**
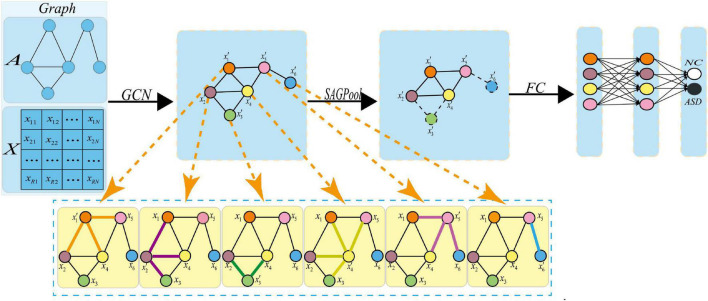
Pooling operation. Where *x*_i_ represents the feature vector of the *i-th* node, $x_{\text{i}}^{\prime }$represents the feature vector of the *i-th* new node obtained after the graph convolution. After the pooling operation, a new graph is obtained, in which the dotted line indicates that the corresponding node should be discarded.

To better reflect the hierarchical structure of the input data and reduce the learning parameters for higher computation efficiency, we add the self-attention pooling operation after the graph convolution. The network architecture is shown in Figure 4. The updating formulas of node feature matrix and adjacency matrix are given by equation (4):


(4) $$\left({\hat{A}}^{\left(l+1\right)},X^{\left(l+1\right)}\right)=R\,e\,L\,u\,\left(S\,A\,G\,P\,o\,o\,l\,\left(G\,C\,N\,\left({\hat{A}}^{\left(l\right)},X^{\left(l\right)}\right)\right)\right)$$

**FIGURE 5:**
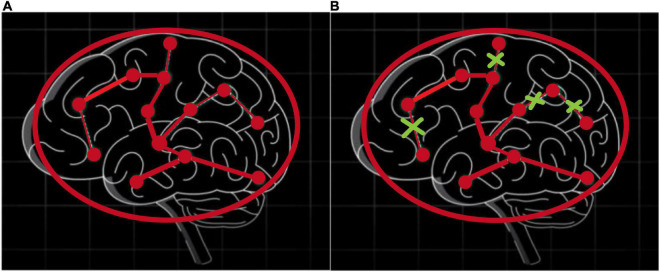
Self-attention graph pooling method diagram, where **(A)** represents the connectivity among brain regions before the pooling operation, **(B)** represents the connectivity among brain regions after the pooling operation, and thickness of lines represents the strength of connectivity among brain regions, the fork sign represents that the pooling operation can discard some less important nodes and retain the nodes with more discriminative features.

To understand the pooling operations in the graph network, Figure 5 shows the changes in brain connectivity before and after the pooling, where thickness of lines represents the strength of connectivity among brain regions, and the fork sign represents that the pooling operation can discard some less important nodes and retain the nodes with more discriminative features. From Figure 5, self-attention graph pooling method cannot only use relatively few parameters to learn hierarchical representation in end-to-end manner, but also use self-attention to distinguish among nodes that should be deleted and retained. SA-GCN not only considers the node features, but also reflects the topology of the graph, which is conducive to improve the accuracy of downstream classification task.

### Construction of Multi-Level Graph Network

**FIGURE 6:**
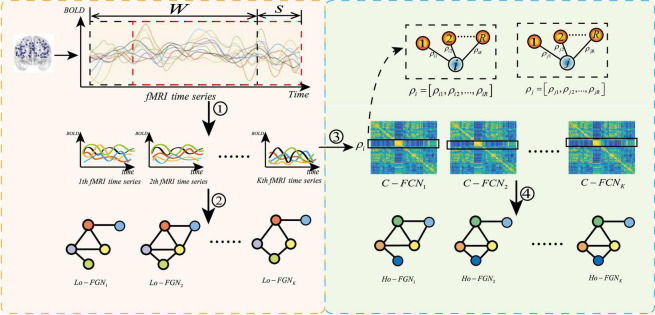
Construction of multi-level function graph network, where ➀ represents get K sub fMRI time series; ➁ represents the construction of Lo-FGN from fMRI time series; ➂ represents the use of Pearson correlation to build a functional connectivity network (FCN); ➃ represents the construction of Ho-FGN from FCN.ρ_i_ represents Pearson correlation between the *i-th* ROI and other ROIs.*C*−*FCN*_i_ represents the *i-th* traditional functional connectivity network.

Feature extraction based on GCN requires the construction of function graph network from fMRI data. The complete function graph network includes two parts: node feature matrix and adjacency matrix. Conventional methods ignore the complementarity of features among different levels. Our method constructs the function graph network from multiple levels, as shown in Figure 6, where the left part is the construction process of low-order functional graph network (Lo-FGN), and the right part illustrates the construction of high-order functional graph network (Ho-FGN).

#### Construction of Low-Order Functional Graph Network

Let *x*_i_(*l*) and *x*_*j*_(*l*) represent the subsequences of the *i-th* and *j-th* ROI in the *l-th* window, respectively. The correlation between time series is calculated by Pearson correlation to obtain FC, and the FC is thresholded by adjusting parameters to obtain the adjacency matrix of Lo-FGN, that is:


(5) $$A_{\text{Lo}}=\varphi \,\left(c\,o\,r\,r\,\left(x_{\text{i}}\,\left(l\right),x_{\text{j}}\,\left(l\right)\right)\right)={\left(\rho_{\text{ij}}\,\left(l\right)\right)}_{1\leq l\leq K,1\leq i,j\leq R}$$

where φ denotes a thresholding operation.

To capture the temporal changes in the original BOLD in the brain area and avoid the timing structure of rs-fMRI is being destructed, we take the mean and variance of the original data *X*_*roi*_ as the node features *X*_*Lo*_ of Lo-FGN as:


(6) $$X_{L\,o}=\left(m\,e\,a\,n\,\left(X\right),v\,a\,r\,\left(X\right)\right)$$

#### Construction of High-Order Functional Graph Network

To characterize the organizational features of the brain and reflect the functional connectivity interaction mode among multiple ROIs, we explore the connection relationship of edges in the graph network to enhance discrimination ability of node features. Based on the “one-time Pearson correlation,” the high-order function connection (Ho-FC) is obtained based on the idea of “correlation of correlation,” and the Ho-FC is thresholded by adjusting parameters to obtain the adjacency matrix of the Ho-FGN as follows:


(7) $$A_{H\,o}=\varphi \,{\left(\rho \,\left(\rho_{\text{ij}}\,\left(l\right)\right)\right)}_{1\leq l\leq K,1\leq i,j\leq R}$$

To better capture the deep-seated node features, the functional connectivity matrix is used as the node feature matrix of Ho-FGN, that is:


(8) $$X_{H\,o}={\left(\rho_{\text{ij}}\,\left(l\right)\right)}_{1\leq l\leq K,1\leq i,j\leq R}$$

## Experiments Analysis

### Experimental Data

The rs-fMRI dataset used in this article is from the ABIDE database, which consists of 17 international imaging sites (Di Martino et al., 2014). To mitigate data heterogeneity, the rs-fMRI data of NUY site with the largest sample size are selected to verify the feasibility of our proposed method. Specifically, rs-fMRI scanning data of 45 patients with ASD and 47 normal control (NC) subjects were included. The subjects ages are between 7 and 15 years, and there are no excessive head movements in any three directions, displacement less than 1.5 mm or angular rotation less than 1.5°. The detailed demographic information of these subjects is summarized in Table 1. There are no significant differences in age, gender, IQ, diagnostic interview, and diagnostic observation (*p* > 0.05) between the two groups.

**TABLE 1:** Demographic information of the subjects.

Characteristic	ASD	NC	*p*-values
Gender (M/F)	36/9	36/11	0.2135*^a^*
Age (mean ± SD)	11.1±2.3	11.0±2.3	0.773*^b^*
FIQ (mean ± SD)	106.8±17.4	113.3±14.1	0.0510*^b^*
ADI-R (mean ± SD)	32.2±14.3*^c^*	−	−
ADOS (mean ± SD)	13.7±5.0	-	−
FD (mean ± SD)	0.14±0.05	0.15±0.07	0.36*^b^*

*M, male; F, female; FIQ, Full Intelligence Quotient; ADI-R, Autism Diagnostic Interview-Revised; ADOS, autism diagnostic observation schedule.*

*^a^The p-value was obtained by χ2-test.*

*^b^The p-value was obtained by two-sample two-tailed t-test.*

*^c^Two patients do not have the ADI-R score.*

The data acquisition and preprocessing follow a standard pipeline, including head movement, normalization, denoising, and other processes and related parameters, which same as some previous pieces of literature (Murdaugh et al., 2012; Satterthwaite et al., 2013; Yan et al., 2013; Washington et al., 2014; Leung et al., 2015; Lin et al., 2015; Ray et al., 2015; Urbain et al., 2016; Reinhart and Nguyen, 2019). Finally, we use the automatic anatomical marker (AAL) map to divide the brain into 116 brain ROIs and calculate the mean value of rs-fMRI time series of each brain ROI, which is represented by the data matrix *X* ∈ *R*^170×116^ for subsequent experiments. Note that 170 represents the total volume of time images and 116 is the total number of all brain ROIs.

### Evaluation Methodology

To verify the effectiveness of the method, we conducted eight experiments based on rs-fMRI data. In the experiment, ASD and NC are considered as positive and negative classes, respectively. All experiments were evaluated by 10 times of fivefold cross-validation. Specifically, we first divide all subjects into 5 subsets (roughly the same size). Then, we take one subset as the test set and the other four subsets as the training data. This process is repeated 10 times to avoid the deviation of random data division in cross-validation. The classification results of all iterations are averaged and evaluated by six metrics: classification accuracy (ACC), sensitivity or true positive rate (TPR), specificity or true negative rate (TNR), positive predictive value (PPV), negative predictive value (NPV), and F1 score. In addition, we performed the statistical significance test (*t*-test) on the accuracy obtained by seven comparison methods and SA-GCN, and the *p*-values of the test are also listed in Table 2. When the *p*-value is less than 0.05, it indicates that there is a significant difference between the two methods.

**TABLE 2:** ASD classification results with different feature strategies.

Model	ACC (%)	*P*-value	TPR (%)	TNR (%)	PPV (%)	NPV (%)	F1 (%)
*CBN*	65.4 ± 0.01	0.0161498	67.3 ± 0.12	62.7 ± 0.06	59.8 ± 0.01	71.2 ± 0.06	63.0 ± 0.01
*GCN*_*(Lo)*_	68.0 ± 0.01	0.0034533	67.8 ± 0.12	66.5 ± 0.04	67.3 ± 0.01	68.1 ± 0.01	67.0 ± 0.04
*SA*−*GCN*_(*Lo*)_	73.0 ± 0.03	0.0306230	65.0 ± 0.07	81.0 ± 0.01	79.6 ± 0.08	71.0 ± 0.08	69.4 ± 0.04
FCN	72.6 ± 0.02	0.0301950	88.2 ± 0.02	56.0 ± 0.07	64.3 ± 0.05	87.9 ± 0.05	74.0 ± 0.01
*GCN*_*(Ho)*_	74.5 ± 0.01	0.0462659	74.7 ± 0.02	74.4 ± 0.04	76.5 ± 0.03	75.0 ± 0.01	75.0 ± 0.08
*SA*−*GCN*_(*Ho*)_	75.0 ± 0.04	0.0453326	70.1 ± 0.12	77.3 ± 0.05	77.9 ± 0.01	74.0 ± 0.01	72.0 ± 0.04
*GCN*_(*Lo*)_ + *GCN*_(*Ho*)_	77.0 ± 0.01	0.0421363	71.3 ± 0.08	78.5 ± 0.04	77.9 ± 0.02	74.7 ± 0.07	73.6 ± 0.04
*SA*−*GCN*_(*Lo*)_ + *SA*−*GCN*_(*Ho*)_	**79.9 ± 0.03**	**/**	**75.6 ± 0.03**	**78.6 ± 0.04**	**78.7 ± 0.01**	**78.6 ± 0.01**	**76.1 ± 0.04**

*The bold values represent the results of our proposed method under different evaluation metrics.*

#### Influence of Parameters on Feature Extraction

**FIGURE 7:**
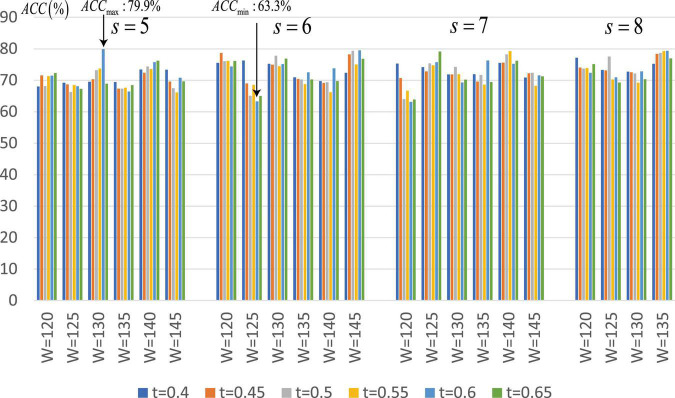
Average classification accuracy (ACC) of SA-GCN with different free parameter combinations (i.e., *W, s*, and *t*).

Since the proposed SA-GCN is a deep learning method, to avoid overfitting, the “sliding window” strategy is adopted to increase the sample size. There are two free parameters, namely, sliding window width (*W*) and translation step size (*s*), which may affect the final classification performance. We set the range of these parameters to *W* ∈ [120,125,130,135,140,145], *s* ∈ [5,6,7,8]. In addition, in the process of constructing Lo-FGN and Ho-FGN, because the brain network is considered to have sparse connection structure, the adjacency matrix is thresholded by adjusting parameters. In the construction of Lo-FGN, the range of threshold *L*_*corr *_is set as *L*_*corr*_ ∈ {(−0.4,0.4),(−0.45,0.45),…,(−0.65,0.65)}. In the construction of Ho-FGN, the range of threshold *H*_corr_ is set as *H*_corr_ ∈ {(−0.4,0.4),(−0.45,0.45),…,(−0.65,0.65)}. To check the influence of threshold *L*_*corr*_ and *H*_*corr*_ on the results, we make *t* = *L*_corr _ = *H*_corr_ for comparative experiment, as shown in Figure 7.

From Figure 7, we have the following conclusions: (1) The classification performance is quite sensitive to free parameters, so it is necessary to continuously adjust parameters to obtain the best performance. We can see that when *W* = 130,*s* = 5,*L*_*corr*_ = *H*_*corr*_ = 0.6, the maximum value of ACC is 79.9%, and when *W* = 125,*s* = 6,*L*_*corr*_ = *H*_*corr*_ = 0.6, the minimum value of ACC is 63.3%; (2) Different thresholds determine different network topologies, which can provide different useful information for ASD identification and obtain different classification performances.

#### Comparison for Autism Spectrum Disorder Diagnosis Using Different Feature Extraction

To verify the effectiveness of the proposed method, we set *W* = 130,*s* = 5,*t* = *L*_*corr*_ = *H*_*corr*_ = 0.6 and conducted extensive experimental comparison based on the following eight methods. Table 2 shows the average classification performance of the above eight methods. Among them, the conventional brain network (CBN) represents the use of the mean and variance of the time series of rs-fMRI as the characteristics;*GCN*_(*Lo*)_indicates that the constructed *Lo*−*FGN*is sent into the GCN network architecture;*SA*−*GCN*_(*Lo*)_indicates that *Lo-FGN* is sent into the GCN network architecture with self-attention pooling operation; FCN represents the characteristics of traditional FC network based on Pearson correlation; “ + ” denotes the fusion operation and the other expressions of similarity.

From Table 2, we can draw three conclusions: (1) The feature extraction using GCN architecture is superior to the traditional feature extraction methods, indicating that GCN can enhance the node features through the connection among nodes, and has strong feature extraction ability; (2) the GCN with pooling operation *via* self-attention mechanism can take into account node features and network topology structure and extract more discriminative features; (3) for Lo-FGN and Ho-FGN, the performance of feature extraction and feature layer fusion based on SA-GCN achieves the best performance, indicating that the effectiveness of feature fusion.

### The Most Distinguishing Features in Autism Spectrum Disorder Diagnosis

To further analyze the pooling operation in GCN with self-attention mechanism, we fed the test datasets into the SA-GCN architecture and counted the probability of occurrence of each node in the remaining nodes after the pooling operation of all test sets scored based on the self-attention mechanism to rank the nodes’ importance, as shown in Table 3.

**TABLE 3:** The 10 most discriminating features and their frequency of occurrence.

*SA*−*GCN*_(*Lo*)_		*SA*−*GCN*_(*Ho*)_	
ROI	Probability of occurrence	ROI	Probability of occurrence
VIIB-Cb.R	0.7717	INS.L	0.7554
VIIB-Cb.L	0.7704	PUT.L	0.7337
HIP.R	0.7663	SFGmed.R	0.73505
II-Cb.R	0.7649	PAL.L	0.7269
VIII-Cb.R	0.7541	PAL.R	0.71875
II-Cb.L	0.7527	PUT.R	0.7174
VIII-Cb.L	0.7412	THA.L	0.7119
I-Cb.L	0.72146	THA.R	0.7119
PreCG.L	0.7269	SFGmed.L	0.70106
THA.R	0.7228	INS.R	0.649

**FIGURE 8:**
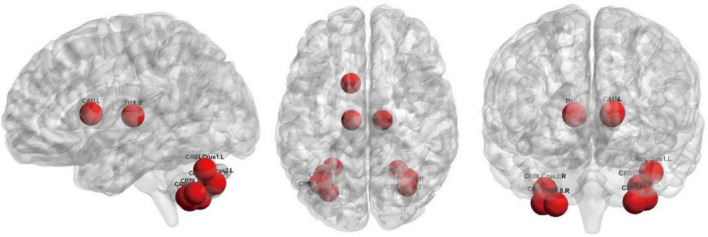
The top 10 nodes (ROIs) of the Lo-FGN screened by the SA-GCN architecture.

The top 10 nodes (ROIs) of the Lo-FGN screened by the SA-GCN architecture are VIIB-Cb.R, VIIB-Cb.L, HIP.R, II-Cb.R, VIII-Cb.R, II-Cb.L, VIII-Cb.L, I-Cb.L, PreCG.L, and THA.R, as shown in Figure 8. Some studies have shown that all these brain regions are associated with ASD.

**FIGURE 9:**
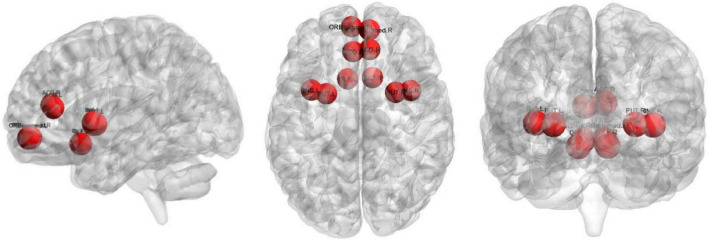
The top 10 nodes (ROIs) of the Ho-FGN screened by the SA-GCN architecture.

The top 10 nodes (ROIs) of the Ho-FGN filtered by the SA-GCN architecture are INS.L, PUT.L, SFGmed.R, PAL.L, PAL.R, PUT.R, THA.L, THA.R, SFGmed.L, and INS.R, as shown in Figure 9. It has been shown that there are significant differences between autistic and normal individuals in SFGmed and INS; SFGmed belongs to the DMN, which is widely believed to play an important role in higher cognitive functions, and abnormalities in the DMN can be observed in a range of neurological disorders (Murdaugh et al., 2012; Washington et al., 2014); INS is highly associated with communication and affective deficits in ASD (Leung et al., 2015; Urbain et al., 2016). In summary, our proposed method can extract deeper and more discriminative features.

## Conclusion

In this article, we propose a novel multi-view feature enhancement method based on SA-GCN. Multi-view discriminative features are extracted from the constructed Lo-FGN and Ho-FGN based on SA-GCN, respectively, and feature layer fusion enables the model to achieve the best classification results. The experimental results show that (1) with the “sliding window” strategy, the sample size can be effectively expanded to avoid the overfitting problem; (2) compared with the other methods, the pooling operation in GCN with self-attention mechanism can extract deeper and more discriminative features, which can help to explore disease-related information for ASD diagnosis; (3) complementary information among features can be achieved from multiple perspectives to improve the disease identification rate.

Finally, SA-GCN can be easily extended for diagnosis of other highly heterogeneous neurodevelopmental disorders, such as Alzheimer’s disease, and depressive illness. Of course, the findings of this study are still preliminary and require further study in the future. As for future work, we plan to extend SA-GCN to other modalities in brain connectomics.

## Data Availability Statement

Publicly available datasets were analyzed in this study. This data can be found here: http://fcon_1000.projects.nitrc.org/indi/abide/abide_I.html.

## Author Contributions

FZ: conceptualization, methodology, and writing-review and editing. NL: conceptualization, software, writing-original draft, methodology, formal analysis, investigation, and validation. HP: validation. XC, YL, HZ, NM, and DC: writing-review and editing. All authors contributed to the article and approved the submitted version.

## Conflict of Interest

The authors declare that the research was conducted in the absence of any commercial or financial relationships that could be construed as a potential conflict of interest.

## Publisher’s Note

All claims expressed in this article are solely those of the authors and do not necessarily represent those of their affiliated organizations, or those of the publisher, the editors and the reviewers. Any product that may be evaluated in this article, or claim that may be made by its manufacturer, is not guaranteed or endorsed by the publisher.
